# Supplementary material for: SARS-CoV-2 transmission dynamics in Belarus in 2020 revealed by genomic and incidence data analysis
Source: Commun Med (Lond). 2021 Sep 16;1:31. doi: 10.1038/s43856-021-00031-1 (PMC9053244; doi:10.1038/s43856-021-00031-1)
Supplement: Supplementary file 1 — Supplementary Information [file 43856_2021_31_MOESM1_ESM.pdf]

SARS-CoV-2 transmission dynamics in Belarus in 2020 revealed by genomic and incidence data analysis.

Alina Nemira[1], Ayotomiwa Ezekiel Adeniyi[1], Elena L. Gasich[3], Kirill Y. Bulda[3], Leonid N. Valentovich[4], Anatoly G. Krasko[3], Olga Glebova[1], Alexander Kirpich[2,*], and Pavel Skums[1,*,°]

[1]Department of Computer Science, Georgia State University, Atlanta, Georgia, USA
[2]Department of Population Health Sciences, School of Public Health, Georgia State University, Atlanta, Georgia, USA
[3]Republican Research and Practical Center for Epidemiology and Microbiology, Minsk, Belarus
[4]Institute of Microbiology, National Academy of Sciences of Belarus, Minsk, Belarus

Supplemental Information.

Keywords: COVID-19, SARS-CoV-2, Belarus, genomic epidemiology phylodynamics, effective reproduction number

° pskums@gsu.edu

* The authors contributed equally.

1 Supplemental Information

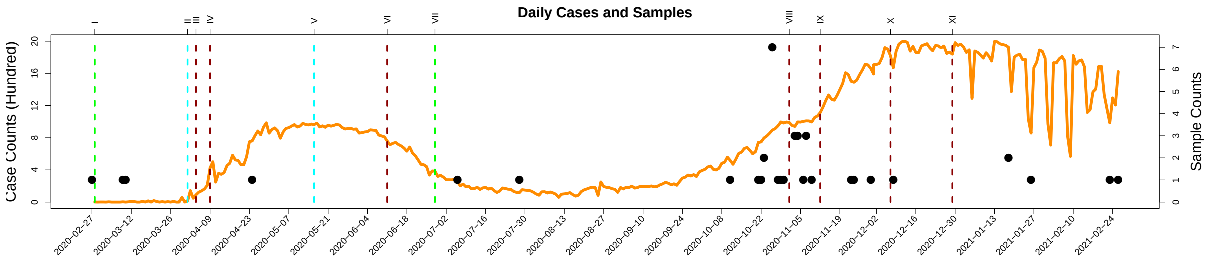

Figure S1: Incidence curve (orange line and numbers of sequenced samples (black dots)) together with major epidemiological events (vertical lines). The boundaries of time intervals for phylodynamic and incidence/testing data analysis are highlighted in green and blue, respectively. The following events are depicted: (I) first reported COVID-19 case. Phylodynamic analysis start date. (II) First detailed numbers of conducted tests are published. Incidence/testing data analysis start date. (III) Spring breaks in schools are extended. (IV) A mandatory 14-day self-isolation for individuals who were arriving from abroad and for close contacts of infected individuals is established. (V) Officially reported peak of the first wave. Incidence/testing data analysis end date. (VI) Self-isolation requirements for persons arriving from selected countries is lifted. Foreign citizens entering Belarus are required to provide a PCR test certificate. (VII) Officially reported statistics meets WHO criterion for the epidemic that is considered to be "under control". The phylodynamic analysis end date. (VIII) Borders are closed for most categories of foreign citizens. (IX) Mask regimen is introduced in the capital city of Minsk. (X) Borders are closed for Belarusian citizens. (XI) Start of mass vaccination is announced.

**Model**	**Parameter**	**Prior distribution**
Strict clock	Clock rate	Gamma(2.56,3200)
HKY	Kappa<br>Gamma shape	Lognormal(1,1.25)<br>Exponential(1,0)
Birth-death skyline	$\mathcal{R}_e$<br>Uninfectious rate<br>Sampling proportion<br>Time of origin	Lognormal(0.8,0.5)<br>Fixed to 36.5 per year<br>Beta(1,99999)<br>Normal(0.89,0.01) for cluster 1,<br>Normal(0.919,0.01) for cluster 2

Table S1: BDSKY model parameters used for $\mathcal{R}_e$ estimation.

	Before NPIs	After NPIs
Australia [6]	1.63	0.48
Russia (hospital settings) [3]	3	1.76
Russia (hospital settings) [3]	3.64	1.85
New Zealand [2]	7	0.2
Israel [4]	2.1	0.525
France [1]	2.56	1.38
France [5]	3	
Germany [5]	1.75	
Italy [5]	2.4	
**Belarus** (this study)	**1.95**	**1.59**

Table S2: Estimations for the effective reproduction number $\mathcal{R}_e$ for different countries reported in the literature.

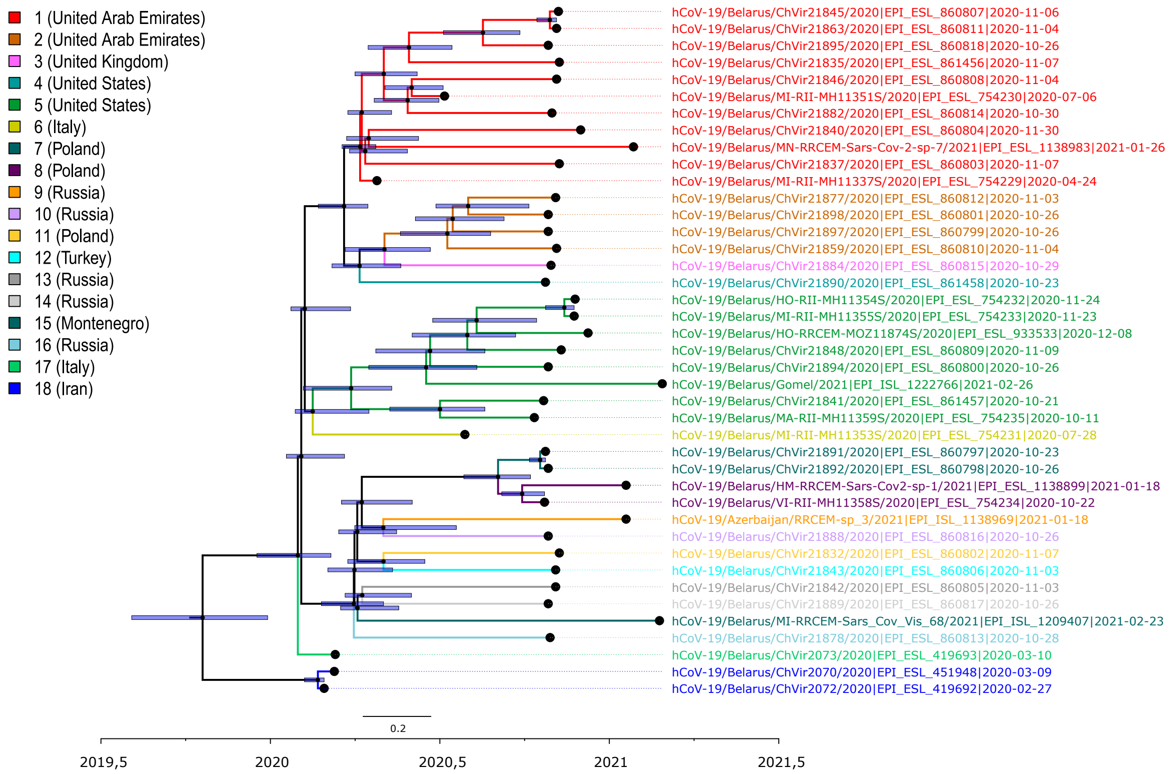

Figure S2: The annotated maximum clade credibility tree with sequence names and visualized 95 HPD in blue: clusters/local lineages numbered from one to eighteen, tree branches and sequence names color-coded by cluster IDs; cluster sources added in parentheses.

Cluster	Sequence	GISAID accession ID	Country
Cluster 1	Ukraine/203100356/2020—2020-05-28	EPI_ISL_512635	Ukraine
	Ukraine/203100318/2020—2020-06-23	EPI_ISL_512598	
	Ukraine/203100333/2020—2020-06-24	EPI_ISL_512612	
	Ukraine/203100319/2020—2020-06-23	EPI_ISL_512599	
	Ukraine/203100335/2020—2020-06-26	EPI_ISL_512615	
	Ukraine/203100336/2020—2020-06-23	EPI_ISL_512615	
	Ukraine/Rivne55/2020—2020-06-04	EPI_ISL_979972	
	Ukraine/ChVir23535_80/2021—2021-01-10	EPI_ISL_1112335	
	Ukraine/ChVir23535_23/2021—2021-01-10	EPI_ISL_1112303	
	Ukraine/203100348/2020—2020-05-16	EPI_ISL_512627	
	Ukraine/Vinnytsia_12/2020—2020-05-27	EPI_ISL_979970	
	Ukraine/203100339/2020—2020-07-11	EPI_ISL_512618	
	Ukraine/ChVir23535_35/2021—2021-01-12	EPI_ISL_1112311	
	Ukraine/203100320/2020—2020-06-23	EPI_ISL_512600	
Cluster 2	Ukraine/203100361/2020—2020-04-24	EPI_ISL_512640	Ukraine
	Ukraine/Kharkiv-877/2020—2020-08-07	EPI_ISL_582510	
	Ukraine/Kharkiv-782/2020—2020-07-31	EPI_ISL_582513	
	Ukraine/Kharkiv-705/2020—2020-07-28	EPI_ISL_576146	
	Ukraine/ChVir23535_53/2021—2021-01-11	EPI_ISL_1112314	
	Ukraine/ChVir23535_21/2021—2021-01-09	EPI_ISL_1112301	
	Ukraine/ChVir23535_77/2021—2021-01-09	EPI_ISL_1112332	
	Ukraine/ChVir23535_79/2021—2021-01-09	EPI_ISL_1112334	
	Ukraine/ChVir23535_42/2021—2021-01-12	EPI_ISL_1112312	
	Ukraine/ChVir23535_29/2021—2021-01-11	EPI_ISL_1112307	
	Ukraine/ChVir23535_3/2021—2021-01-11	EPI_ISL_1112293	
	Ukraine/ChVir23535_51/2021—2021-01-12	EPI_ISL_1112313	
	Ukraine/Kharkiv-879/2020—2020-08-07	EPI_ISL_582511	
	Ukraine/ChVir2353_54/2021—2021-01-11	EPI_ISL_1112294	
Cluster 1	hCoV-19/Belarus/MN-RRCEM-Sars-Cov-2-sp-7/2021—2021-01-26	EPI_ISL_1138983	Belarus
	Belarus/MI-RII-MH11337S/2020—2020-04-24	EPI_ISL_754229	
	Belarus/MI-RII-MH11351S/2020—2020-07-06	EPI_ISL_754230	
	Belarus/ChVir21882/2020—2020-10-30	EPI_ISL_860814	
	Belarus/ChVir21837/2020—2020-11-07	EPI_ISL_860803	
	Belarus/ChVir21840/2020—2020-11-30	EPI_ISL_860804	
	Belarus/ChVir21846/2020—2020-11-04	EPI_ISL_860808	
	Belarus/ChVir21895/2020—2020-10-26	EPI_ISL_860818	
	Belarus/ChVir21845/2020—2020-11-06	EPI_ISL_860807	
	Belarus/ChVir21863/2020—2020-11-04	EPI_ISL_860811	
	Belarus/ChVir21835/2020—2020-11-07	EPI_ISL_861456	
Cluster 5	hCoV-19/Belarus/Gomel/2021—2021-02-26	EPI_ISL_1222766	Belarus
	Belarus/HO-RII-MH11354S/2020—2020-11-24	EPI_ISL_754232	
	Belarus/MI-RII-MH11355S/2020—2020-11-23	EPI_ISL_754233	
	Belarus/MA-RII-MH11359S/2020—2020-10-11	EPI_ISL_754235	
	Belarus/ChVir21894/2020—2020-10-26	EPI_ISL_860800	
	Belarus/ChVir21848/2020—2020-11-09	EPI_ISL_860809	
	Belarus/ChVir21841/2020—2020-10-21	EPI_ISL_861457	
	Belarus/HO-RRCEM-MOZ11874S/2020—2020-12-08	EPI_ISL_933533	

Table S3: Analyzed sequences and their sampling times.

Cluster name	Sequence Name	Calendar Date Time of MRCA	Number of Sequences
1	Belarus/MN-RRCEM-Sars-Cov2-sp-7<br>Belarus/ChVir21835<br>Belarus/ChVir21863<br>Belarus/ChVir21845<br>Belarus/ChVir21895<br>Belarus/ChVir21846<br>Belarus/ChVir21882<br>Belarus/MI-RII-MH11351S<br>Belarus/ChVir21840<br>Belarus/ChVir21837<br>Belarus/MI-RII-MH11337S	April 5, 2020	11
2	Belarus/ChVir21877<br>Belarus/ChVir21898<br>Belarus/ChVir21897<br>Belarus/ChVir21859	July 9, 2020	4
3	Belarus/ChVir21884	May 4, 2020	1
4	Belarus/ChVir21890	April 4, 2020	1
5	Belarus/HO-RII-MH11354S<br>Belarus/MI-RII-MH11355<br>Belarus/HO-RRCEM-MOZ11874S<br>Belarus/ChVir21848<br>Belarus/ChVir21894<br>Belarus/ChVir21841<br>Belarus/MA-RII-MH11359S<br>Belarus/Gomel/2021	March 28, 2020	8
6	Belarus/MI-RII-MH11353S	February 13, 2020	1
7	Belarus/ChVir21891<br>Belarus/ChVir21892	October 19, 2020	2
8	Belarus/VI-RII-MH11358S<br>Belarus/HM-RRCEM-Sars-CoV2-sp-1	September 27, 2020	2
9	Azerbaijan/RRCEM-sp_3/2021	April 30, 2020	1
10	Belarus/ChVir21888	April 30, 2020	1
11	Belarus/ChVir21832	April 30, 2020	1
12	Belarus/ChVir21843	April 30, 2020	1
13	Belarus/ChVir21842	April 8, 2020	1
14	Belarus/ChVir21889	April 8, 2020	1
15	Belarus/MI-RRCEM-Sars_Cov_Vis_68/2021	April 5, 2020	1
16	Belarus/ChVir2073	January 30, 2020	1
17	Belarus/ChVir21878	April 8, 2020	1
18	Belarus/ChVir2072<br>Belarus/ChVir2070	February 21, 2020	2

Table S4: Inferred clusters and their times of MRCA.

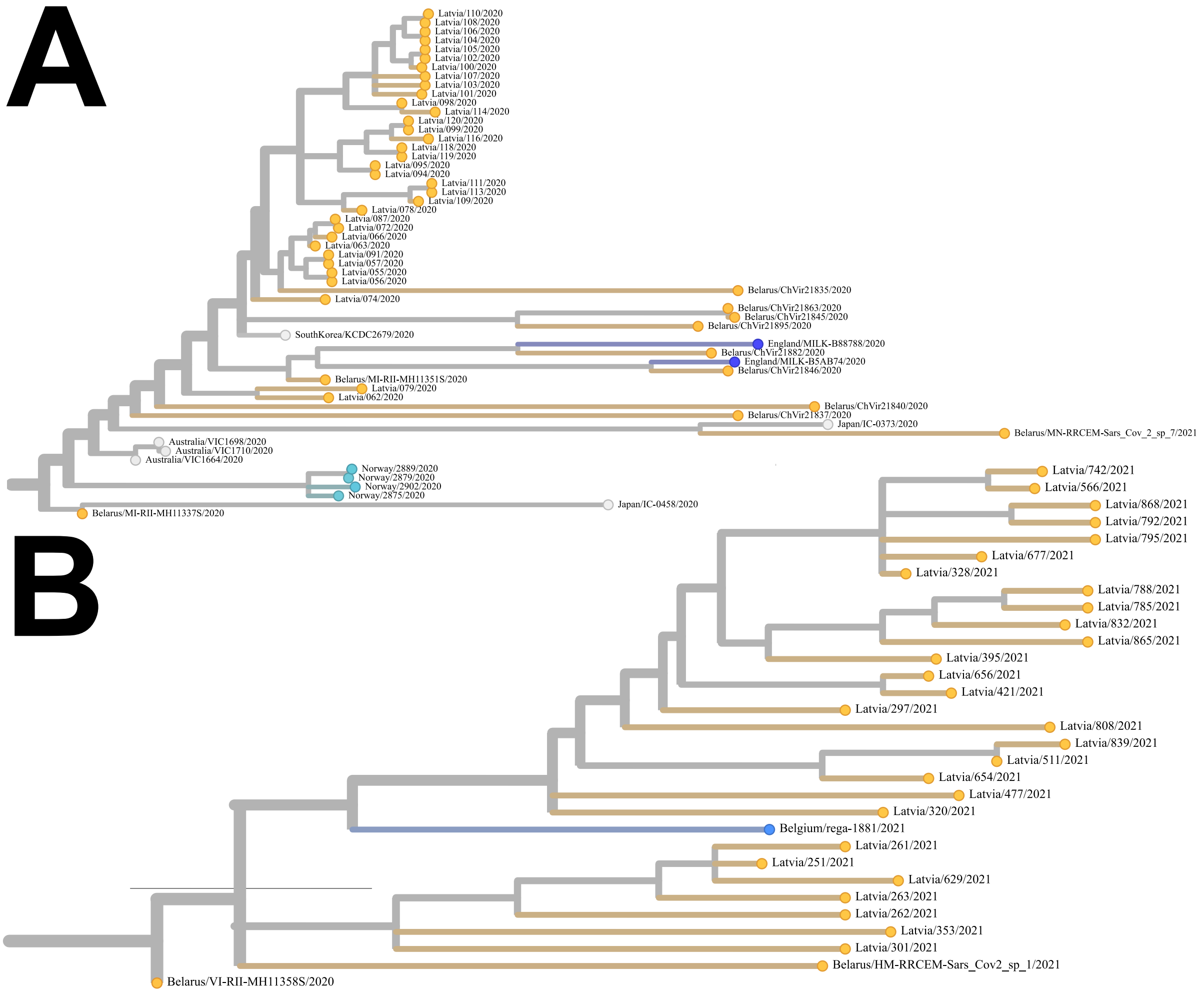

Figure S3: Latvian lineages originated from two alleged introductions from Belarus. Trees were visualized by Nextstrain

Supplementary References

[1] Gonché Danesh, Baptiste Elie, Yannis Michalakis, Mircea T Sofonea, Antonin Bal, Sylvie Behillil, Grégory Destras, David Boutolleau, Sonia Burrel, Anne-Geneviève Marcelin, et al. Early phylodynamics analysis of the covid-19 epidemic in france. *medRxiv*, 2020.

[2] Jemma L Geoghegan, Xiaoyun Ren, Matthew Storey, James Hadfield, Lauren Jelley, Sarah Jefferies, Jill Sherwood, Shevaun Paine, Sue Huang, Jordan Douglas, et al. Genomic epidemiology reveals transmission patterns and dynamics of sars-cov-2 in aotearoa new zealand. *Nature communications*, 11(1):1–7, 2020.

[3] Andrey B Komissarov, Ksenia R Safina, Sofya K Garushyants, Artem V Fadeev, Mariia V Sergeeva, Anna A Ivanova, Daria M Danilenko, Dmitry Lioznov, Olga V Shneider, Nikita Shvyrev, et al. Genomic epidemiology of the early stages of the sars-cov-2 outbreak in russia. *Nature communications*, 12(1):1–13, 2021.

[4] Danielle Miller, Michael A Martin, Noam Harel, Omer Tirosh, Talia Kustin, Moran Meir, Nadav Sorek, Shiraz Gefen-Halevi, Sharon Amit, Olesya Vorontsov, et al. Full genome viral sequences inform patterns of sars-cov-2 spread into and within israel. *Nature communications*, 11(1):1–10, 2020.

[5] Sarah A Nadeau, Timothy G Vaughan, Jérémie Sciré, Jana S Huisman, and Tanja Stadler. The origin and early spread of sars-cov-2 in europe. *Proceedings of the National Academy of Sciences*, 118(9), 2021.

[6] Torsten Seemann, Courtney R Lane, Norelle L Sherry, Sebastian Duchene, Anders Gonçalves da Silva, Leon Caly, Michelle Sait, Susan A Ballard, Kristy Horan, Mark B Schultz, et al. Tracking the covid-19 pandemic in australia using genomics. *Nature communications*, 11(1):1–9, 2020.
